# Development of a Heat-Shock Inducible Gene Expression System in the Red Alga *Cyanidioschyzon merolae*

**DOI:** 10.1371/journal.pone.0111261

**Published:** 2014-10-22

**Authors:** Nobuko Sumiya, Takayuki Fujiwara, Yusuke Kobayashi, Osami Misumi, Shin-ya Miyagishima

**Affiliations:** 1 Center for Frontier Research, National Institute of Genetics, Mishima, Shizuoka, Japan; 2 Japan Science and Technology Agency, CREST, Kawaguchi, Saitama, Japan; 3 Department of Biological Science and Chemistry, Faculty of Science, Yamaguchi University, Yamaguchi, Yamaguchi, Japan; 4 Department of Genetics, Graduate University for Advanced Studies (SOKENDAI), Mishima, Shizuoka, Japan; Louisiana State University Health Sciences Center, United States of America

## Abstract

The cell of the unicellular red alga *Cyanidioschyzon merolae* contains a single chloroplast and mitochondrion, the division of which is tightly synchronized by a light/dark cycle. The genome content is extremely simple, with a low level of genetic redundancy, in photosynthetic eukaryotes. In addition, transient transformation and stable transformation by homologous recombination have been reported. However, for molecular genetic analyses of phenomena that are essential for cellular growth and survival, inducible gene expression/suppression systems are needed. Here, we report the development of a heat-shock inducible gene expression system in *C. merolae*. *CMJ101C*, encoding a small heat shock protein, is transcribed only when cells are exposed to an elevated temperature. Using a superfolder GFP as a reporter protein, the 200-bp upstream region of *CMJ101C orf* was determined to be the optimal promoter for heat-shock induction. The optimal temperature to induce expression is 50°C, at which *C. merolae* cells are able to proliferate. At least a 30-min heat shock is required for the expression of a protein of interest and a 60-min heat shock yields the maximum level of protein expression. After the heat shock, the mRNA level decreases rapidly. As an example of the system, the expression of a dominant negative form of chloroplast division DRP5B protein, which has a mutation in the GTPase domain, was induced. Expression of the dominant negative DRP5B resulted in the appearance of aberrant-shaped cells in which two daughter chloroplasts and the cells are still connected by a small DRP5B positive tube-like structure. This result suggests that the dominant negative DRP5B inhibited the final scission of the chloroplast division site, but not the earlier stages of division site constriction. It is also suggested that cell cycle progression is not arrested by the impairment of chloroplast division at the final stage.

## Introduction

The unicellular red algae Cyanidiales are defined as thermoacidophiles because they grow at an extremely low pH (0.05–5) and relatively high temperature (35–56°C) [1]. Among the Cyanidiales, the unicellular red alga, *Cyanidioschyzon merolae*, contains just one nucleus, mitochondrion and chloroplast per cell [2]. Cell division and organelle division are tightly synchronized by the light/dark cycle [3]. These features make this alga a model organism for studying the mechanisms of chloroplast and mitochondrial division [4]. In addition, the complete determination of the nuclear, mitochondrion and chloroplast genomes [5], [6], [7], [8], as well as the establishment of genetic manipulation methods, such as transient plasmid expression [9] and gene targeting by homologous recombination [10], [11], [12], have contributed to the growing interest in *C. merolae* as a model organism. The *C. merolae* nuclear genome size is 16.5 Mbp with 4,774 protein-coding genes. The genome content is extremely simple, with a low level of redundancy in photosynthetic eukaryotes. In addition, most of the genes lack introns [7], [8], [13]. Thus, in addition to studies on chloroplast and mitochondrial division, *C. merolae* has become a promising organism for the study of cell biology and metabolisms in photosynthetic eukaryotes, such as vacuolar inheritance [14], [15], the dynamics of the endoplasmic reticulum [16] and Golgi apparatus [17], nitrogen assimilation [18], [19], chromosome organization [20], and circadian rhythms [21].

The simple nature of the cellular architecture and genome content of *C. merolae* has facilitated the characterization of fundamental intracellular phenomena. However, since such phenomena are essential to cellular growth and survival, inactivation or modification of the mechanisms by gene manipulation would likely be lethal. To overcome this critical issue, analyses of primary defects using conditional mutations, an approach which is not effective under permissive environmental conditions but can be effective under certain tightly restricted conditions, is greatly desired.

To this end, we have developed an inducible gene expression system using a heat-shock promoter in *C. merolae*, which we report in this study. As an example of an application of the system, expression of a dominant negative form of the DRP5B/CmDnm2 protein was induced by heat shock, after which the effect on chloroplast division and cell cycle progression was examined.

## Materials and Methods

### Algal culture

*Cyanidioschyzon merolae* 10D or stable transformants were grown in 50 mL of Allen's medium in a 100 mL test tube under continuous light (100 µE m^−2^ s^−1^) with aeration (500 mL ambient air min^−1^) [22].

### Semi-quantitative RT-PCR

Cells were harvested by centrifugation at 800×*g*, frozen in liquid nitrogen and stored at −80°C until use. Total RNA was extracted with the RNeasy Mini Kit and RNase-Free DNase set (Qiagen, Venlo, Netherlands). cDNA was synthesized from the total RNA using 6 nucleotide random primers with ThermoScript RT (Life Technologies, Carlsbad, CA). The primers used for semi-quantitative RT-PCR are listed in Table S1.

### DNA construction for the superfolder GFP (sfGFP) expressing strain

According to the amino acid sequence of original sfGFP [23], the oligonucleotides encoding sfGFP for the expression in *C. merolae* were artificially synthesized so as to introduce the mutations S30R, Y39N, Q80R, F99S, N105T, Y145F, M153T, V163A, I171V, A206V and L231H (accession no. AB971579) into the nucleotide sequence encoding the enhanced GFP (EGFP; [24]). For further construction, *Bam*HI and *Not*I sites were fused to the 5′- and 3′- terminals of the sequence, respectively.

To produce a strain which constitutively expresses sfGFP, a plasmid containing *APCC* promoter-*sfGFP*-*URA* was constructed by eliminating the *E2F orf* from the plasmid *APCC* promoter-*sfGFP*-*pmE2F*-*URA* [21]. The regions other than *pmE2F* were amplified from the original plasmid by the primers sfGFP720taaRbt and btUTR(+1), then circularized using an In-Fusion cloning kit (Takara Bio, Shiga, Japan).

To prepare a URA (the URA5.3 gene; CMK046C) expressing strain as a control, *APCC* promoter-*EGFP* was eliminated from the plasmid containing *APCC* promoter-*EGFP*-URA (pD184-O250-EGFP-URA_Cm-Cm_; [11]). The regions other than *APCC* promoter-*EGFP* were amplified from the original plasmid using the primers d184(+25)Rura d184(+25)R and CmUra(−897)F and the fragment was circularized using an In-Fusion cloning kit. The primer sequences for the construction are listed in Table S1. The plasmid pD184-O250-EGFP-URA_Cm-Cm_ [11] was used to produce the strain constitutively expressing EGFP.

### Construction of the plasmids for the transient transformation assays

To transiently express the sfGFP driven by the upstream region of *C. merolae CMJ101C* from a plasmid, we constructed plasmids in which a series of different lengths of the *CMJ101C* upstream region were fused to the *sfGFP orf* and the *Nos* terminator. *Bam*HI- and *Not*I-digested *sfGFP* was cloned between the *Bam*HI and *Not*I sites of the pI050P-GFP vector [25] to produce the pI050P-sfGFP plasmid. A series of different length of the upstream region of *CMJ101C* (−1000 to +2, −750 to +2, −500 to +2, −345 to +2, −250 to +2, −200 to +2 and −100 to +2) were amplified by PCR. For the forward primers, 101_−1000_Xba, 101_−750_Xba, 101_−500_Xba, 101_−345_Xba, 101_−250_Xba, 101_−200_Xba and 101_−100_Xba were used. For the single reverse primer, 101_+2_*Bgl*II was used. The amplified fragments were digested with *Xba*I and *Bgl*II and cloned between the *Xba*I and *Bam*HI sites of pI050P-sfGFP. For convenience, the resultant plasmids were named P-1000, P-750, P-500, P-345, P-250, P-200, and P-100, respectively.

### Construction of the plasmids for the stable transformants

To prepare the stable transformants which express sfGFP by 200, 250, or 345-bp upstream region of *CMJ101C orf*, upstream region comprising *CMJ101C*, *sfGFP* and the *nos* terminator was amplified with the primers CMD184D∼+25-M13F_Inf_1 and M13 R. The amplified products were replaced with *APCC* promoter-*EGFP* in pD184-O250-EGFP-Ura_Cm-Cm_ [11] by ligation with a linearized vector that was amplified from the plasmid pD184-O250-EGFP-Ura_Cm-Cm_ by the primers CMD184C_+25R and M13R_CmUra-897∼Inf_1 using In-Fusion cloning kit.

To produce the GFP-DRP5B or GFP-DRP5B K135A expressing the stable transformants, the stop codon of *sfGFP* in pI050P-sfGFP was eliminated. The promoter region of the resultant plasmid pI050P-sfGFP (-stop) was then substituted with a 200-bp upstream region of *CMJ101C*. The 200-bp upstream region of *CMJ101C* was amplified with the primers 101_−200_Xba and 101_+2_*Bgl*II. The amplified fragment was digested with *Xba*I and *Bgl*II and cloned between the *Xba*I and *Bam*HI sites of pI050P-sfGFP (-stop). The resultant plasmid was digested with *Not*I and *C. merolae DRP5B orf* (*CMN262C*), which was amplified with the primers GFP_Not_Dynamin_inf_1 and GFP_Not_Dynamin_inf_2, was inserted by the In-Fusion cloning kit. For the cloning of the dominant negative form of *DRP5B*, Lys-135 was replaced by Ala using the primers Dynamin_K44A_sense and Dynamin_K44A antisense. The primer sequences for the construction are listed in Table S1.

### Transformation of *C. merolae*

Transient transformation was performed using a polyethylene glycol (PEG)-mediated protocol, as described previously [9]. To produce stable transformants, 6 µg of the PCR product obtained by the primers D184(1270)F and D184(+1448)R from the aforementioned plasmids were introduced via the PEG-mediated protocol into *C. merolae* M4, which has a mutation in the *URA* gene [10], and the transformants were selected as described previously [19].

### Quantitative PCR Analyses

Quantitative-PCR analyses were performed using a StepOnePlus Real-Time PCR System (Life Technologies) and a 20 µl reaction mixture containing 2 µl template DNA, 0.25 nM primers and 10 µl Power SYBR Green Master Mix (Life Technologies). To examine copy number of the introduced DNA fragment, standard curves were constructed using serially diluted solutions of DNA isolated from CmsfGFP-1 strain cells and the relevant sets of primers. The value of *GFP* (from 638 to 717 bp in the *orf*) in each strain was normalized with the data of a region (from 741 to 841 bp in *CMD184C orf*) located outside of the introduced DNA fragment and then the copy number was displayed relative to an EGFP strain, which contains ∼1 copy of *EGFP* sequence [11]. To examine cell cycle progression of the heat-shocked culture, standard curves were constructed by using serially diluted solutions of cDNA that was prepared from S-200 cells cultured at 42°C under light with the relevant sets of primers. The value of *PCNA* (from 468 to 524 bp in the *orf*) and *CDC20* (from 419 to 473 bp in the *orf*) were normalized with the data of DRP3 (from 1981 to 2050 bp in the *orf*). The primer sequences for the quantitative-PCR analyses are shown in Table S1.

### Immunoblot analyses

5×10^8^ cells were harvested by centrifugation at 800×*g* for 5 min and stored at −80°C. Cells were suspended in SDS-PAGE sample buffer (50 mM Tris, pH 6.8, 6% 2-mercaptoethanol, 2% sodium dodecyl sulfate, 10% glycerol) and sonicated on ice for 6 cycles (10 s on and 30 s off at 310 W; Bioruptor UCW-310, Cosmobio, Tokyo, Japan). After centrifugation at 15,000×*g* for 10 min at 4°C, the supernatant fraction was separated by SDS-PAGE and analyzed by immunoblotting with a mouse monoclonal anti-GFP antibody (Living Colors A.v. Monoclonal Antibody (JL-8), Clontech, Mountain View, CA) as the primary antibody at a dilution of 1∶1,000 and goat anti-mouse IgG-HRP antibody (Life Technologies) as the secondary antibody at a dilution of 1∶2,000.

### Fluorescence microscopy

The cells were examined using an epifluorescence microscope (BX51, Olympus, Tokyo, Japan) equipped with a CCD camera (DP70, Olympus). The GFP fluorescence and autofluorescence of chlorophyll were captured using an Olympus WIB cube.

### DAPI (4′, 6-diamidino-2-phenylindole) staining

Cells were harvested by centrifugation at 2,000×*g* for 3 min and fixed in 1% glutaraldehyde in TAN (20 mM Tris-HCl, pH 7.6, 0.5 mM EDTA, 1.2 mM spermidine, 0.6 mM spermin, 7 mM 2-mercaptoethanol, 0.4 mM phenylmethyl- sufonyl fluoride). The cells were stained with 1.0 µg ml^−1^ DAPI by pressing them between a slide glass and a cover slip.

## Results and Discussion

### Expression pattern of *CMJ101C* mRNA

To develop an inducible gene expression system in *C. merolae* using a heat-shock promoter, we searched for *C. merolae HSP* genes that are transcribed only when cells are exposed to elevated temperature (i.e. with no basal level expression at a lower temperature).

**Figure 1:**
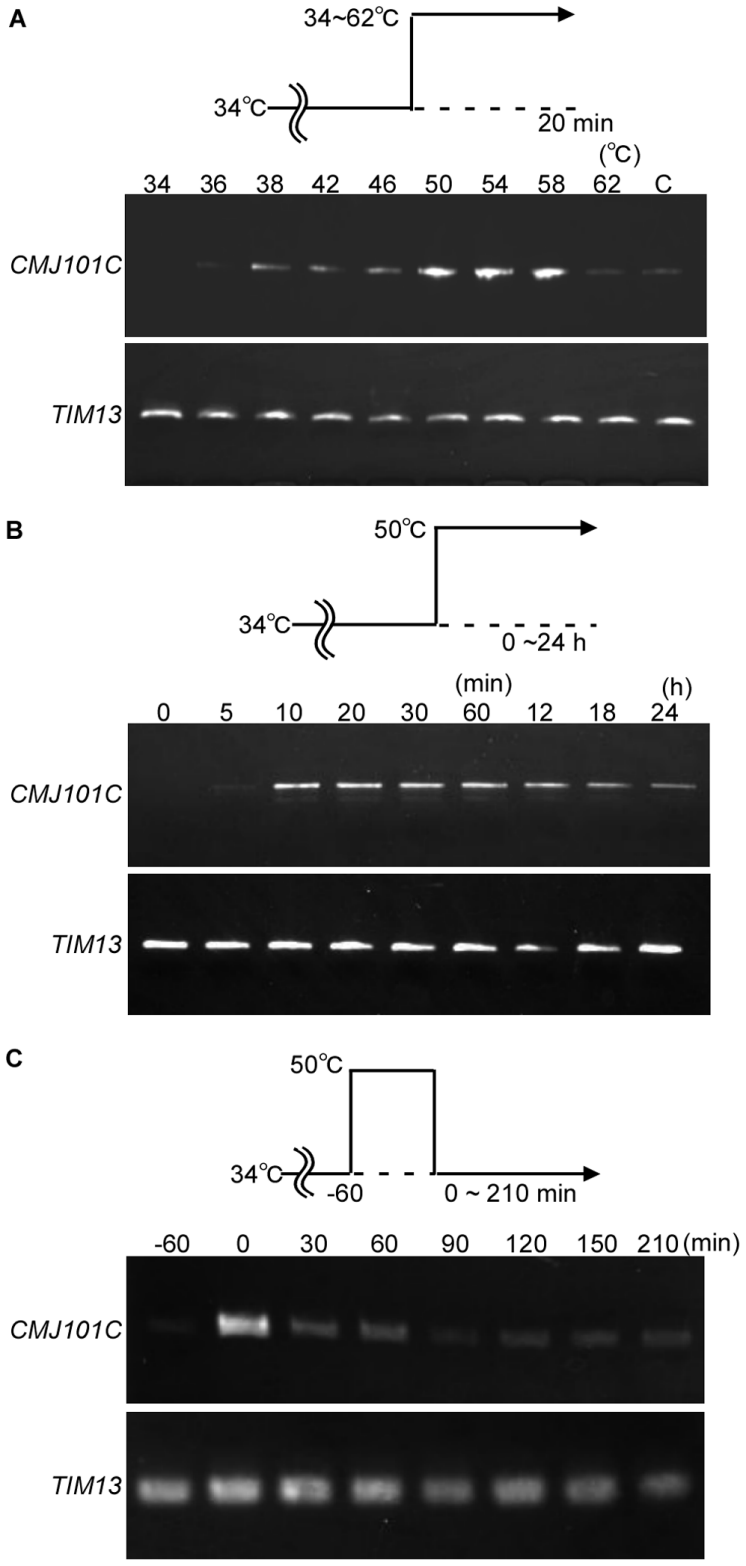
Patterns of *CMJ101C* mRNA induced expression by heat shock. (A) The expression pattern of *CMJ101C mRNA* after a 20 min heat shock at 34∼62°C. (B) The expression pattern of *CMJ101C mRNA* after a 0∼24 hr heat shock at 50°C. (C) *C. merolae* wild-type cells were cultured at 34°C before the heat shock. The expression pattern of *CMJ101C mRNA* before and 0∼210 min after one-hour heat shock at 50°C. mRNA levels were examined by semi-quantitative RT-PCR. *TIM13* (mitochondrial intermembrane space complex subunit, *CMB148C*) was used as a quantitative control. C in (A) represents the mRNA level in the cells cultured at 42°C continuously.

In the *C. merola*e genome database (http://merolae.biol.s.u-tokyo.ac.jp/), 14 genes are predicted to encode heat shock proteins. Among these 14 genes, the transcripts of 13 were found in the previous expressed sequence tag (EST) data that was obtained from the culture at the optimal temperature (42°C), while the transcript of one gene, *CMJ101C* (A BLASTP search in the NCBI database showed that *CMJ101C* encodes a member of the Hsp20/alpha crystallin family of proteins) that was not found in the EST data. To examine whether *CMJ101C* is transcribed only at higher temperatures, *C. merolae* cells cultured at 34°C for one day were shifted to 34∼62°C for 20 min. By RT-PCR analyses, *CMJ101C* mRNA was detected at only 38°C or higher (Figure 1A) and the mRNA level exhibited a maximum at 50°C.

To determine the optimum duration of the heat shock for inducing *CMJ101C* transcription, the cells were shifted from 34°C to 50°C. The mRNA level increased for 10 min after the temperature shift and the level was maintained for 24 hr (Figure 1B). After the 60-min heat shock, the *CMJ101C* mRNA level rapidly decreased over 30 min (Figure 1C), while a marginal transcript level was detected for at least 210 min, suggesting that *CMJ101C* transcription ceased immediately after the end of the heat shock. These results led us to explore the usage of the *CMJ101C* promoter for an inducible gene expression system in *C. merolae*.

### sfGFP functions as a reporter protein in *C. merolae*

**Figure 2:**
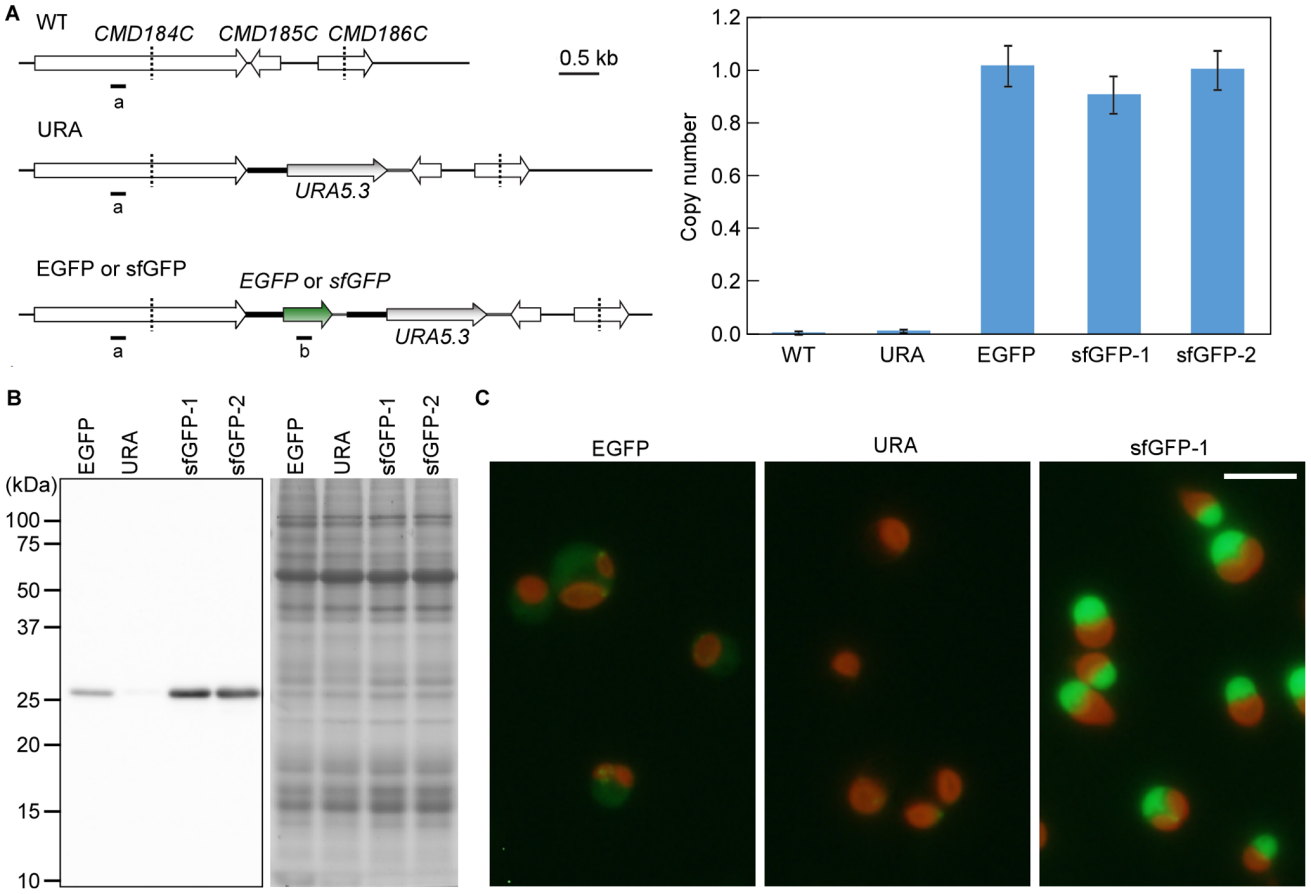
Comparison of the protein level and fluorescent intensity between EGFP and sfGFP expressed from the *C. merolae* genome. A single copy of *EGFP* or *sfGFP* was constitutively expressed by the *APCC* promoter from a *C. merolae* genomic-neutral locus. (A) Quantitative PCR analyses showing that a single copy of *EGFP* or *sfGFP* was integrated into the nuclear genome of the respective transformants. The CMD184C-mid region, which locates outside of the introduced DNA fragment (a) and GFP sequence (b), was investigated by quantitative PCR. The value of (b) in each strain was normalized against that of (a) to estimate the copy number. The copy number is shown relative to an *EGFP* strain, which contains ∼1 copy of the *EGFP* sequence [11]. The bars indicate the SD (*n* = 3). (B) EGFP and sfGFP protein levels were compared by immunoblotting. Two sfGFP strains were isolated independently (sfGFP-1, sfGFP-2). The URA strain was used as a negative control, in which the *URA* gene (*URA5.3*, *CMK046C*) was introduced into the genomic-neutral locus of *C. merolae* M4 that has a mutation in the *URA5.3* gene. Coomassie Brilliant Blue (CBB) staining of the gel is shown as a loading control. (C) Fluorescence of EGFP and sfGFP were observed by fluorescence microscopy. The fluorescence from GFP and autofluorescence of chlorophyll were captured simultaneously. The images were captured using the same exposure time (1 sec). The scale bar  = 5 µm.

To examine the transcriptional activity of the *CMJ101C* promoter, we searched for GFP variants that are appropriate for *C. merolae* culture at higher temperatures (34∼50°C). The folding of enhanced GFP (EGFP) is optimized at 37°C [24]. In contrast, superfolder GFP (sfGFP; [23]) exhibits bright fluorescence in extreme thermophiles, even at 70°C [26]. In order to compare the efficiency of EGFP and sfGFP as reporter proteins in *C. merolae*, we prepared strains that constitutively express EGFP or sfGFP (one copy of the *orf* was integrated into the genome, Figure 2A) under the *C. merolae* constitutive *APCC* promoter from a genomic-neutral locus [11]. By quantitative-PCR analyses, we confirmed that a single GFP sequence was integrated into the genome in the transformants (Figure 2A). Immunoblot analysis with an using the anti-GFP antibody showed that the level of sfGFP was higher than that of EGFP in *C. merolae* cells cultured at 42°C, a temperature which is optimal for *C. merolae* growth (Figure 2B). In addition, fluorescence microscopy revealed that sfGFP-expressing cells exhibited brighter fluorescence than EGFP-expressing cells (Figure 2C). Because the nucleotide sequences encoding the EGFP and sfGFP that we expressed in *C. merolae* do not contain any rare codons of *C. merolae*, the above results suggest that sfGFP is more stable than EGFP in *C. merolae* when the cells were cultured at 42°C. Thus, we used sfGFP as the reporter protein for further investigation.

### Determination of the optimal *CMJ101C* promoter region for the heat-shock-inducible gene expression system

**Figure 3:**
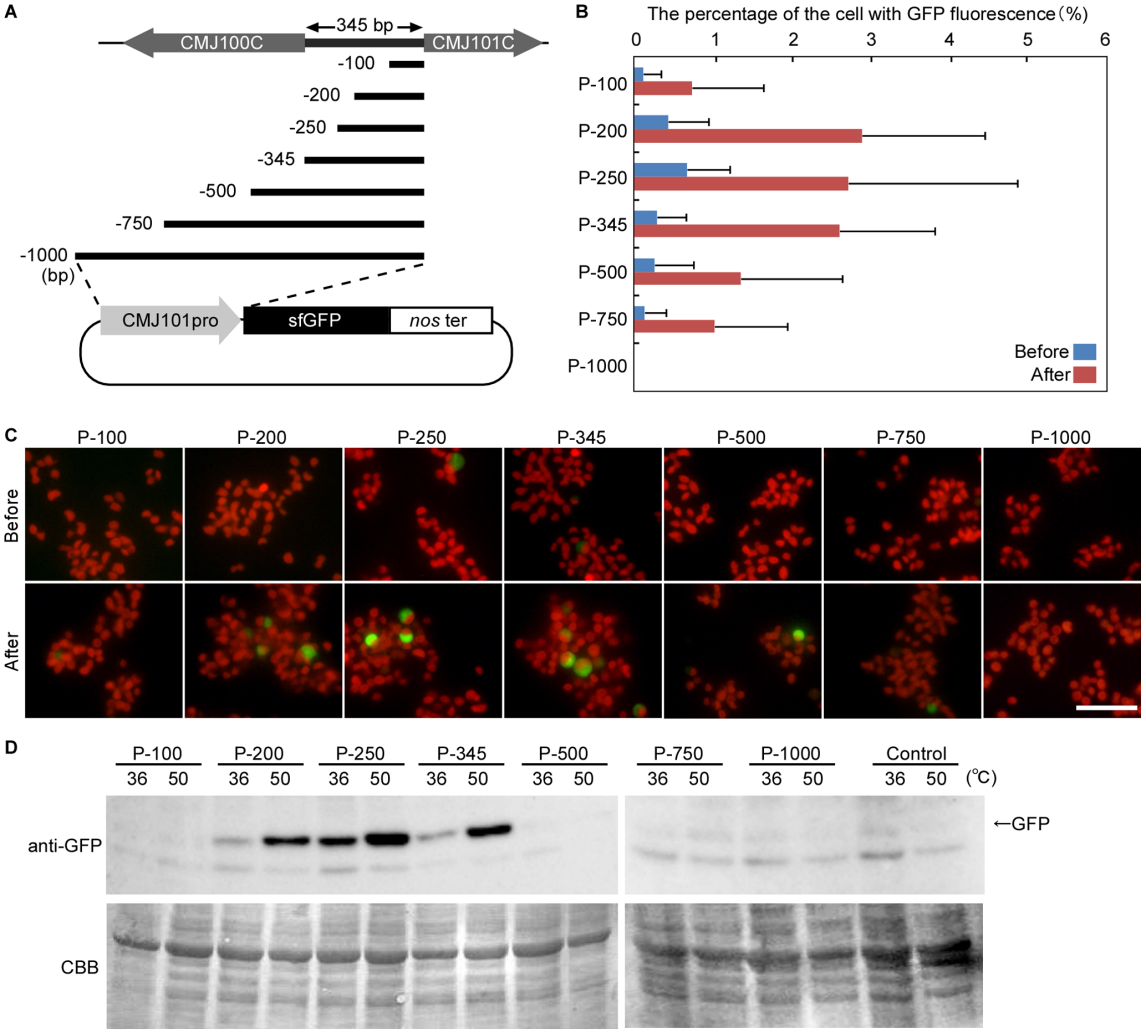
Transcriptional activities of the upstream region of *CMJ101C orf* before and after heat shock treatment in transiently transformed cells. Several different lengths of the upstream region of *CMJ101C orf* were respectively fused with *sfGFP orf*. The plasmids were transiently transformed into *C. merolae* wild-type cells. After transformation, cells were cultured at 36°C for 1 day and then shifted to 50°C for 1 h. Expression of the GFP protein was examined by fluorescence microscopy and immunoblotting. (A) Schematic diagram of the plasmids used. (B and C) The percentage of the cells with GFP fluorescence was compared before and after the 1-h 50°C heat shock treatment. The red is the autofluorescence of chlorophyll. The error bars represent the standard deviation calculated from three individual experiments (*n* = 3). 100 cells were counted for each experiment. The scale bar is 10 µm. (D) Immunoblotting with the anti-GFP antibody before (36°C) and after (50°C) the heat shock treatment. CBB staining of the PVDF membrane is shown as a loading control.

Upstream of the *CMJ101C orf*, the *CMJ100C orf* locates in the opposite orientation and the interval between the two *orfs* is 345 bp (Figure 3A). It is well known that there are conserved *cis*-regulatory promoter elements called heat shock elements in the upstream region of heat shock genes (reviewed in [27]), but there are no typical heat shock elements (at least of the three nGAAn elements) in the 345-bp region. In order to define the region that is optimal for transcription specifically in response to heat shock, we fused a series of *CMJ101C* upstream sequences of different length to *sfGFP orf* and the constructs (plasmids; P-100 to P-1,000) were transiently transformed into *C. merolae* wild-type cells (Figure 3A). The transient transformation of *C. merolae* cells resulted at most in 2∼10% of transformants in a population [9]. After transformation, cells were cultured at 36°C for 24 h and then the temperature was shifted to 50°C.

After 1-h heat shock at 50°C, GFP fluorescence-positive cells were detected, especially in the cultures transformed with P-200, P-250 and P-345. However, the fluorescence-positive cells were also observed before the heat shock treatment in the culture transformed with P-250 (Figures 3B, 3C). Consistent with this observation, the immunoblot analysis showed that GFP was expressed in the cultures transformed with P-200, P-250 and P-345 after 1-h heat shock at 50°C, and that GFP was expressed before the heat shock in the culture transformed with P-250 (Figure 3D).

The above results obtained by the transient expression of GFP from the plasmids suggest that a 200-bp or 345-bp upstream sequence of *CMJ101C orf* is suitable for the heat shock inducible gene expression system, but the plasmid copy number of the transformants varies with the method, so it is difficult to exactly compare the transcriptional activity of the upstream sequences before and after the heat shock. Thus, we prepared the stable transformants (S-200, S-250 and S-345) in which a single promoter (the 200, 250 or 345-bp upstream region of *CMJ101C orf*) and *GFP orf* fusion was inserted into the same genomic-neutral site for further evaluation.

**Figure 4:**
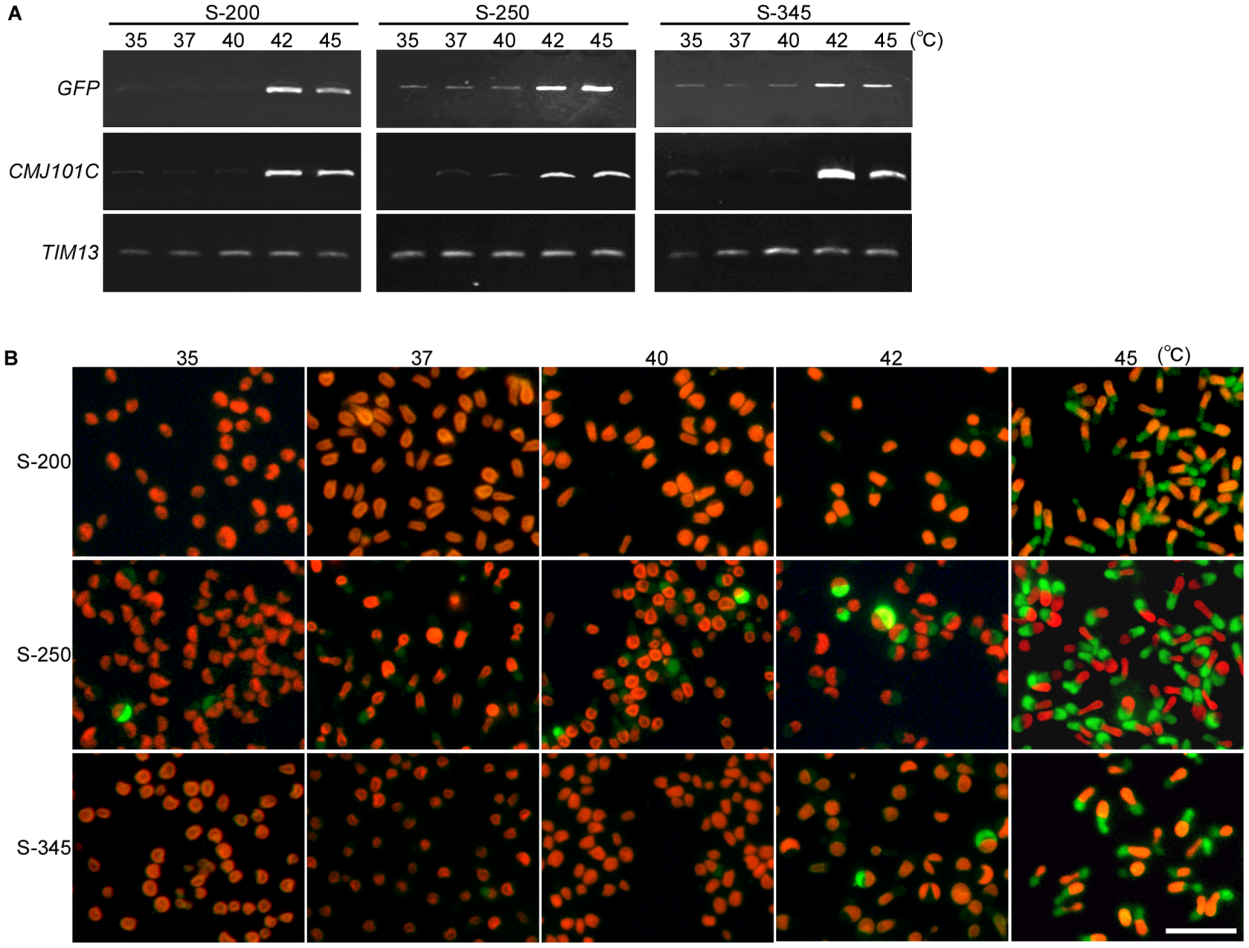
Basal transcriptional activities of the upstream region of *CMJ101C orf* at lower temperatures in the stable transformants. A single copy of *sfGFP orf* fused with the 200 (S-200), 250 (S-250) or 345 (S-345)-bp upstream region of *CMJ101 orf* (Figure 3A) was integrated into the *C. merolae* genomic-neutral locus [11]. The stable transformants were cultured at 35∼45°C for 1 day. (A) Semi-quantitative RT-PCR showing the level of the *sfGFP* mRNA. *CMJ101C* and *TIM13* (*CMB148C*) were used as a positive and quantitative control, respectively. (B) Micrographs showing the GFP fluorescence and autofluorescence of chlorophyll (red). The scale bar is 10 µm.

To evaluate the basal level of GFP expression before the induction by heat shock, the S-200, S-250 and S-345 stable transformants were cultured at 35, 37, 40, 42, and 45°C for one day. It was shown by semi-quantitative RT-PCR that *GFP* mRNA was detected in the S-200 cells at only 42°C and 45°C. In contrast, in the S-250 and S-345 cells, the mRNA was detected at all of the temperatures tested, even though a higher level of mRNA was detected at 42°C and 45°C (Figure 4A). Consistent with the results of RT-PCR, GFP fluorescence was detected in the S-200 cells at only 42°C and 45°C. In contrast, some of the S-250 and S-345 cells exhibited GFP fluorescence at lower temperatures (S-250 at 35, 37 and 40°C, and S-345 at 37 and 40°C)(Figure 4B). The strongest fluorescence was observed at 45°C in all of the transformants. These results indicate that the 200-bp upstream region of *CMJ101C orf* is the most suitable for the heat shock inducible system in the sense of there being little leakage of expression at lower temperatures.

### Determination of the optimum temperature and duration for heat-shock inducible gene expression in the stable transformants

**Figure 5:**
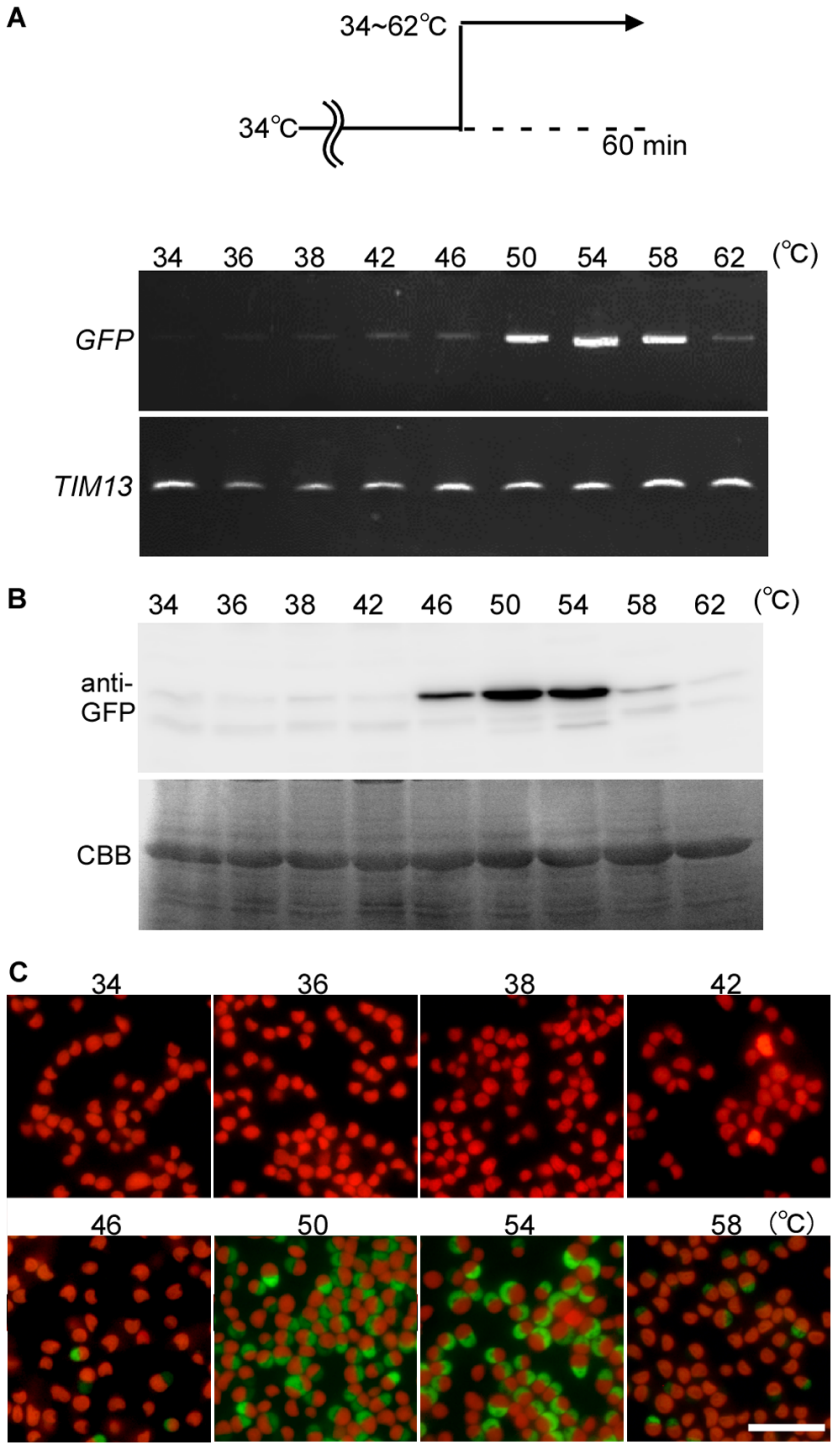
Optimum temperature for heat shock inducible expression in the stable transformants. The stable S-200 transformant (Figure 4) cultured at 34°C was shifted to 34∼62°C and cultured for 60 min. (A) Semi-quantitative RT-PCR showing the *GFP* mRNA level. *TIM13* (*CMB148C*) was used as a quantitative control. (B) Immunoblotting with the anti-GFP antibody showing the GFP protein level. CBB staining of the PVDF membrane is shown as a loading control. (C) Micrographs showing the GFP fluorescence and autofluorescence of chlorophyll (red). The scale bar is 10 µm.

To determine the optimum temperature at which heat shock induces gene expression in the stable transformants, the S-200 cells were shifted from 34°C to 34–62°C and cultured for one hour. The semi-quantitative RT-PCR showed that the *GFP* mRNA level peaked at 50–58°C (Figure 5A). Immunoblotting and fluorescence microscopy showed that the GFP protein and fluorescent level peaked at 50–54°C (Figure 5B, 5C). Based on these results, we fixed the heat shock temperature at 50°C for further analyses.

**Figure 6:**
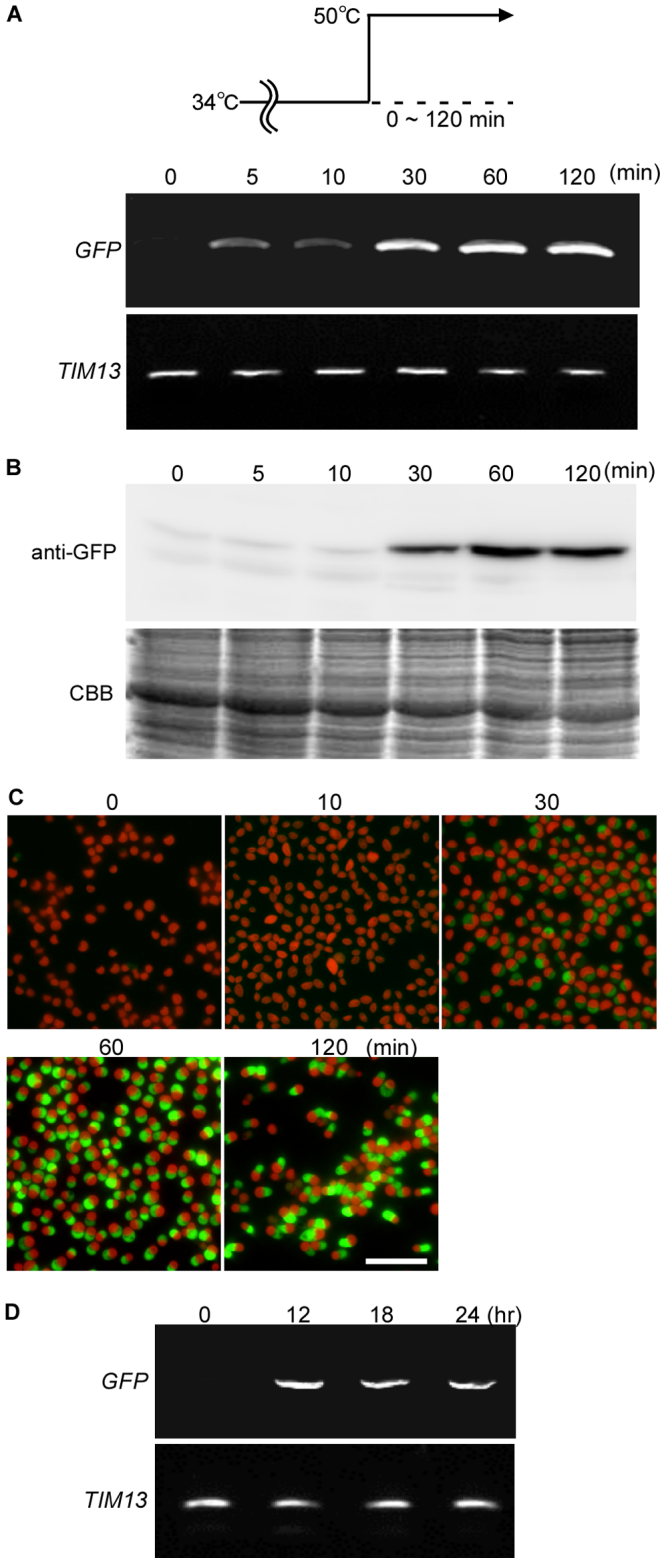
Duration-dependent effect of 50°C heat shock on the mRNA and protein levels in the stable transformants. The stable S-200 transformant (Figure 4) cultured at 34°C was shifted to 50°C and cultured for 120 min or 24 h. (A) Semi-quantitative RT-PCR showing the *GFP* mRNA level. *TIM13* (*CMB148C*) was used as a quantitative control. (B) Immunoblotting with the anti-GFP antibody showing the GFP protein level. CBB staining of the PVDF membrane is shown as a loading control. (C) Micrographs showing the GFP fluorescence and autofluorescence of chlorophyll (red). The scale bar is 10 µm. (D) Semi-quantitative RT-PCR showing the *GFP* mRNA level up to 24 h at 50°C. *TIM13* (*CMB148C*) was used as a quantitative control.

To determine the optimum duration of the 50°C heat shock to induce gene expression in the stable transformants, the S-200 cells were shifted from 34°C to 50°C and cultured for 120 min. *GFP* mRNA was detected in within 5 min of the temperature shift and reached the maximum level at 30 min (Figure 6A). The GFP protein was detected at 30 min and reached the maximum level at 60 min (Figure 6B). GFP fluorescence-positive cells were detected at 30 min and the strongest fluorescence was detected at 60 min (Figure 6C). Thus, for the expression of GFP, at least 30-min heat shock is required and the protein expression level increased for a further 60 min after the temperature shift. Because *GFP* mRNA was detected for at least 24 hours in cells cultured at 50°C (Figure 6D), the duration of translational induction is likely to be able to be extended for particular purposes.

**Figure 7:**
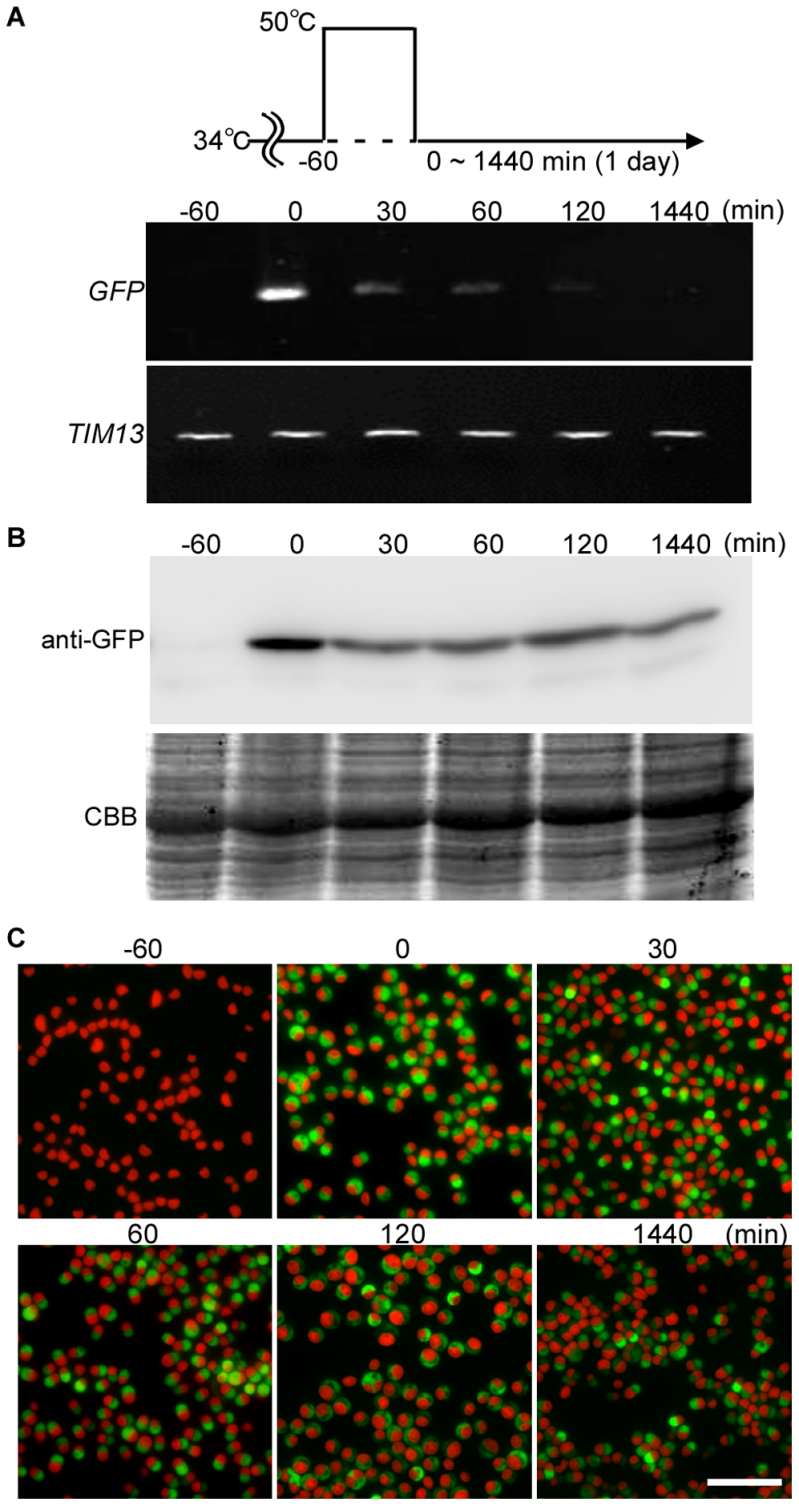
Change in the levels of *GFP* mRNA and GFP protein after 1-h 50°C heat shock in the stable transformants. The stable S-200 transformant (Figure 4) cultured at 34°C (−60 min) was shifted to 50°C for 1 h (0 min) and then returned to 34°C. (A) Semi-quantitative RT-PCR showing the *GFP* mRNA level. *TIM13* (*CMB148C*) was used as a quantitative control. (B) Immunoblotting with the anti-GFP antibody showing the GFP protein level. CBB staining of the PVDF membrane is shown as a loading control. (C) Micrographs showing the GFP fluorescence and autofluorescence of chlorophyll (red). The scale bar is 10 µm.

After the one hour heat shock at 50°C, the *GFP* mRNA level decreased rapidly (Figure 7A). In contrast, the GFP protein and fluorescence was detected even one day after the heat shock (Figures 7B, 7C). This difference between the mRNA and protein level is probably because of the high stability of GFP [28]. In conclusion, for the expression of a target gene by the 200-bp upstream region of *CMJ101C orf,* a heat shock at 50°C for at least 30 min is the optimum condition for achieving inducible protein expression in *C. merolae*.

### Effect of DRP5B mutation on chloroplast division and cell cycle progression: An example of the application of the *C. merolae* heat-shock gene expression system

To apply the inducible gene expression system to an investigation of chloroplast division, we expressed a dominant negative form of DRP5B/CmDmm2 which has a mutation in the GTPase domain. Because the *C. merolae* cell, as in the case of many other algal species, contains only a single chloroplast, the blockage of chloroplast division is probably lethal and thus conditional mutation, such as the inducible expression system employed in this study, is required to characterize the effect of the mutation on chloroplast division.

**Figure 8:**
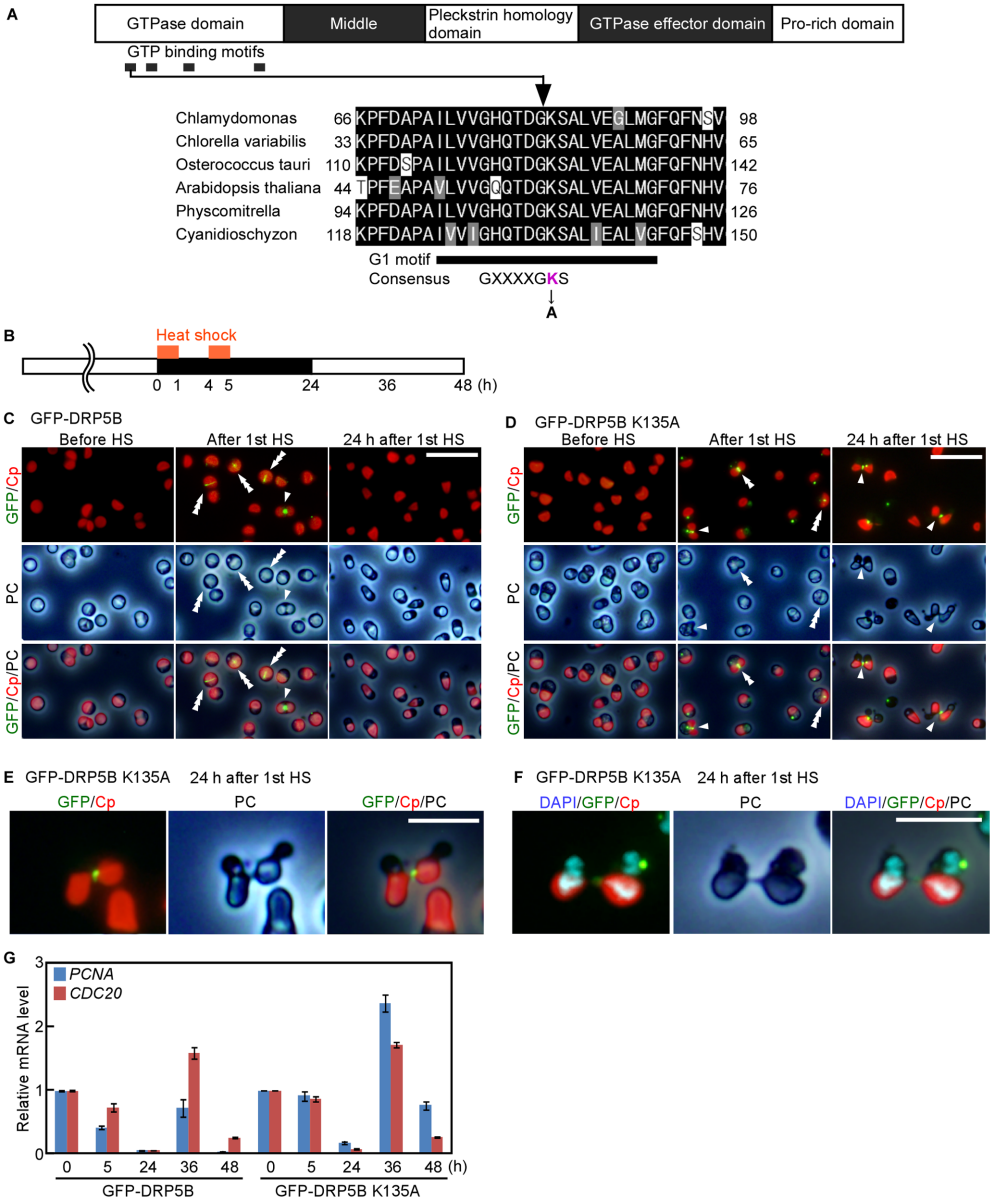
Effect of the DRP5B K135A dominant negative mutation on chloroplast division and cell cycle progression in *C. merolae*. (A) Schematic diagram of the domain composition of the conventional dynamin protein which is involved in endocytosis. In the GTPase domain, four GTP binding motifs (G1, G2, G3 and G4) contribute to the GTP binding. Partial alignment of the DRP5B proteins showed the conservation of the G1 motif. The DRP5B amino acid sequences (*Chlamydomonas reinhardtii*, GI: 30349146; *Chlorella variabilis*, GI: 552813628; *Ostreococcus tauri*, GI: 308803420; *Arabidopsis thaliana*, GI: 30349146; *Physcomitrella patens*, GI: 224434564; *Cyanidioschyzon merolae*, GI: 544214467) were aligned using ClustalW [35], [36]. Alanine substitution for the conserved lysine residue within G1 of human dynamin 1 is known to result in a dominant negative effect by abolishing GTP-binding activity [32]. (B to F) GFP-DRP5B or GFP-DRP5B K135A cells cultured at 42°C under light were transferred to dark condition to stop cell growth and new entrance into S phase. Then cells were heat-shocked at 50°C for 1 h x two times. After cultivation under dark for 12 h, cells were transferred to light condition to resume cell growth (B). GFP fluorescence (along with the red the autofluorescence of chlorophyll) phase-contrast (PC) images are shown (C, GFP-DRP5B, D and E, GFP-DRP5B K135A). The triple arrowheads indicate the chloroplast before division site constriction. The double arrowheads indicates the chloroplast in the earlier stage of division site constriction. The arrowhead indicates the chloroplast in the final stage of division. Images by DAPI staining (blue fluorescence) for GFP-DRP5B K135A 24 h after the heat shock are also shown in (F). The scale bars represent 10 µm in (C, D) and 5 µm in (E, F). (G) Quantitative RT-PCR analyses showing the change in the transcript levels of an S-phase marker *PCNA* (*CMS101C*) and an M-phase marker *CDC20* (*CMA138C*) in the GFP-DRP5B and GFP-DRP5B K135A expressing cells. *DRP3* was used as the internal control. The expression levels at 0 h were defined as 1.0. The bars indicate the standard deviation (*n* = 3).

DRP5B is a member of a eukaryotic dynamin family of self-assembling GTPases and localizes on the cytosolic side of the chloroplast division site, where it is involved in the division process [29], [30]. Conventional dynamin, which is involved in endocytosis, consists of five domains: an N-terminal GTPase domain, a middle domain, a pleckstrin homology domain, a GTPase effecter domain and a proline-rich domain (Reviewed in [31], Figure 8A). GTPase activity is essential for membrane fission in endocytosis and the K44A mutation in human dynamin 1 is known to abolish the activity of dynamin binding GTP, and the expression of the K44A mutant protein exhibits dominant-negative effects [32]. The GTP binding domain is well conserved in the DRP5B of algae and plants (Figure 8A). It has been shown that DRP5B is required for normal chloroplast division based on the impairment of chloroplast division in DRP5B knockout or null mutants in *Arabidopsis thaliana* [30] and *Physcomitrella patens* [33]. In addition, DRP5B is recruited to the chloroplast division site before the onset of division site constriction and persists there throughout the division process [29]. However, it is not known how GTP-binding and/or GTP hydrolysis by DRP5B is involved in chloroplast division. To investigate the role of GTP-binding and/or hydrolysis by DRP5B in chloroplast division in *C. merolae*, we induced the expression of GFP-DRP5B or GFP-DRP5B K135A, the mutation of which corresponds to K44A of the human dynamin1 mutation, by the heat shock system in the stable transformants (Figure 8A).

In exponentially proliferating culture of both of the transformants, the GFP signal was not detected at 40°C. The cells were transferred to a dark condition to stop cell growth and new entrance of G1-phase cells into the S phase and then were heat-shocked at 50°C for 1 h (Figure 8B). After the heat shock, the GFP signal was detected at the chloroplast division site (Figures 8C, 8D. indicated with single, double and triple arrowheads), although additional fluorescent speckles were observed in the cytosol of the GFP-DRP5B K135A expressing cells (Figure 8D). Cells were heat-shocked again for 1 h (4–5 h in Figure 8B). After 24-h incubation under dark at 42°C, GFP-DRP5B cells had completed chloroplast division and subsequent cytokinesis, and thus GFP fluorescence was not detected. In contrast, GFP fluorescence was still observed in some GFP-DRP5B K135A cells (Figure 8D). After 1-h heat shock, GFP fluorescence was observed at the chloroplasts division site before and during the earlier and later stages of the division site constriction in the both transgenic lines. In contrast, after 24-h incubation under dark, GFP-DRP5B K135A cells with GFP fluorescence were at the final stage of chloroplast division and displayed aberrant shapes in which two daughter chloroplasts and the cells were still connected by a small DRP5B K135A positive tube-like structure (Figures 8D, E, indicated by the arrowheads in D). DNA-specific fluorescent dye (DAPI) -staining showed that these aberrant shaped cells contain two daughter nuclei on opposite sides of the tube-like structure (Figure 8F). These results suggest that the expression of DRP5B K135A inhibited the final scission of the division site, but not the earlier stage of division site constriction in the course of chloroplast division. It is also suggested that cell cycle progression is not arrested by the impairment of chloroplast division at the final stage, although the completion of cytokinesis is blocked, probably induced by obstruction brought about by the tubular bridge connecting the two daughter chloroplasts.

When GFP-DRP5B and GFP-DRP5B K135A expressing cells were transferred to light condition after 24-h dark period, the transcript levels of both S-phase marker *PCNA* (CMS101C) and M-phase marker *CDC20* (CMA138C) [22] increased for 12 hour (24 h to 36 h in Figure 8G) and then decreased (36 h to 48 h in Figure 8G). These results indicate that both GFP-DRP5B K135A expressing cells entered and finished the next cell cycle even though final stage of chloroplast division and cytokinesis had been blocked.

In conclusion, we developed a heat-shock inducible gene expression system in *C. merolae*. By using the *CMJ101C* promoter, the heat shock at 50°C for 1 h resulted in the induction of maximum level of mRNA and protein expression. In addition, the results show that the level of expression is adjustable by changing the duration or temperature of the administered heat shock.

As an example of an application of the system, here we have examined the function of DRP5B in chloroplast division by expressing the dominant negative form. Although it has not been tested in this study, this system is probably also applicable to the knockdown expression of genes of interest by heat shock induction of antisense RNA expression. The *C. merolae* genome does not encode RNAi machinery components such as Dicer [34]. However, gene expression knockdown by antisense RNA has been achieved by the transient introduction of plasmids in *C. merolae* [25].

The use of the authentic *URA* (*CMK046C*) selection marker guarantees single-copy insertion of constructs into the genome [11], [20]. In contrast, a recent study showed that the use of an artificial *URA* gene results in genomic insertion of multiple copies (5∼50 copies) of a construct into genomic-neutral loci [11]. By combining the heat shock inducible expression system and the artificial *URA* marker, it is likely to be possible to further increase the induction level of mRNA or antisense RNA.

## Supporting Information

Table S1 Sequences of primers used in this study. Restriction enzyme sites are underlined.
(DOCX)
