# Supplementary material for: Development of a Heat-Shock Inducible Gene Expression System in the Red Alga Cyanidioschyzon merolae
Source: PLoS One. 2014 Oct 22;9(10):e111261. doi: 10.1371/journal.pone.0111261 (PMC4206486; doi:10.1371/journal.pone.0111261)
Supplement: Table S1 — Sequences of primers used in this study. Restriction enzyme sites are underlined. (DOCX) [file pone.0111261.s001.docx]

**Table S1**

Sequences of primers used in this study. Restriction enzyme sites are underlined

| Primer name | sequence |
| --- | --- |
| Primers for semi-quantitative RT-PCR | |
| CMJ101C-LEFT | TGCGCTCACTGCAAGGAAAC |
| CMJ101C-RIGHT | CCATCGAGGGCGATCTTGAC |
| CMB148C-LEFT | AACCAAGCGCTACGCTGACC |
| CMB148C-RIGHT | CGACCGTTGCTGTCGCTCTA |
| GFP-RT_LEFT | CGGCAAGCTGACCCTGAAGT |
| GFP-RT_RIGHT | CCAGCAGGACCATGTGATCG |
| Primers for plasmid construction | |
| sfGFP720taaRbt | TAAATAGCTAGTTTACTTGTACAGCTCGTCCATGC |
| btUTR(+1) | TAAACTAGCTATTTATCTGGTACATATCATTCAT |
| D184(+25)Rura 184(+25)R | TTCGCCCCTCAGTTCCGTCACCCTCGGGACTTG |
| CmUra(-897)F | GAACTGAGGGGCGAACGCA |
| D184(1270)F | ACACGAATCACACGGTGCTG |
| D184(+1448)R | TTGCCGATAACGCAGAAGAGA |
| 101_-1000_Xba | GCTCTAGAATCTAGGAAGGGAAACCC |
| 101_-750_Xba | GCTCTAGAGAGCGAACGCTCGAACGCACCA |
| 101_-500_Xba | GCTCTAGAACCACACCGTTGGCCTTGGCCG |
| 101_-345~ Xba | GCTCTAGAGGCTCGCTCACGTTTG |
| 101_-250_Xba | GCTCTAGAGAAACACCAAAGCGTAACAAAG |
| 101_-200_Xba | GCTCTAGACTTATAGCTTACGTGGCGG |
| 101_-100_Xba | GCTCTAGAGGATCGCAGATGCTGAAATACC |
| 101 _+2_*Bgl*II | GAAGATCTGAATCCCTGGTTCTCTC |
| CMD184D~+25-M13F_Inf_1 | GTCCCGAGGGTGACGTGTAAAACGACGGCCAGTG |
| M13 R | CAGGAAACAGCTATGAC |
| CMD184C_+25R | CGTCACCCTCGGGACTTG |
| M13R_CmUra-897~_Inf_1 | CATAGCTGTTTCCTGGAACTGAGGGGCGAACGC |
| GFP_Not_Dynamin_inf_1 | TGTACAAGAGCGGCCGCATGACCGCAAGGCGTTTG |
| GFP_Not_Dynamin_inf_2 | TGCAGCCGGGCGGCCGCCCAGGCCCGTTGAAGGCT |
| Dynamin_K135A_sense | CCAAACGGATGGAgcaTCTGCACTGATTG |
| Dynamin_K135A_antisense | CAATCAGTGCAGAtgcTCCATCCGTTTGG |
| Primers for quantitative PCR | |
| D184(741) | CGATGTGACCTCTCCCGGTGGT |
| D184(821) | TCCCATTTGAAGCGCAAGT |
| GFP(638)Fc | ACGAGAAGCGCGATCACA |
| GFP(717)Rc | CTTGTACAGCTCGTCCATGC |
| PCNA_CMS101C_qRT_F | CTCCGTGCGCATTTCTGTAA |
| PCNA_CMS101C_qRT_R | TCGTCACCACGAACCGTAAA |
| Cdc20_CMA138C_qRT_F | CAGTAGGGACCGCCTTTGG |
| Cdc20_CMA138C_qRT_R | CGCTTGTTCGCTTCAACATC |
| DRP3(1981)F | GCAGGCGAAGTATCGAGC |
| DRP3(2050)R | AGTAACTCGCAAGAAGCGTCT |
